# Predicting the Addition of Information Regarding Clinically Significant Adverse Drug Reactions to Japanese Drug Package Inserts Using a Machine-Learning Model

**DOI:** 10.1007/s43441-023-00603-4

**Published:** 2023-12-22

**Authors:** Takashi Watanabe, Kaori Ambe, Masahiro Tohkin

**Affiliations:** Department of Regulatory Science, Graduate School of Pharmaceutical Sciences, Nagoya City University, 3-1, Tanabe-dori, Mizuho-ku, Nagoya, 467-8603 Japan

**Keywords:** Drug package insert, Pharmacovigilance, Adverse drug reaction, Post-market drug safety, Machine learning

## Abstract

**Purpose:**

To develop a machine learning (ML)-based model for predicting the addition of clinically significant adverse reaction (CSAR)-associated information to drug package inserts (PIs) based on information of adverse drug reaction (ADR) cases during the post-marketing stage in Japan.

**Methods:**

We collected data on CSARs added to PIs from August 2011 to March 2020. ADR cases that led to CSARs resulting in PI revisions were considered as a positive case, and ML was used to construct a binary classification model to predict the PI revisions. We selected 34 features based on the ADR aggregate data collected 6 months before PI revisions. Prediction performance was evaluated using the Matthews correlation coefficient (MCC).

**Results:**

We found CSAR information added to PIs in 617 cases, 334 of which were due to the accumulation of domestic cases, and used only domestic case data for the prediction model. Among prediction models developed using several kinds of algorithms, the support vector machine with the radial basis function kernel with feature selection showed the highest predictive performance, having an MCC of 0.938 for the cross-validation and 0.922 for the test dataset. The feature with the highest importance in the model was the “average number of patients reported per quarter.”

**Conclusion:**

Our model accurately predicted PI revisions using information on ADR cases that occurred 6 months before. This is the first ML model that can predict the necessary safety measures and is an efficient method for guiding the decision to adopt additional safety measures early.

**Supplementary Information:**

The online version contains supplementary material available at 10.1007/s43441-023-00603-4.

## Introduction

The Pharmaceuticals and Medical Devices Agency (PMDA) collects and shares a wide range of post-marketing safety information, including adverse drug reaction (ADR) reports from manufacturers, medical professions, and patients, and information on safety measures taken by foreign regulatory authorities with the Pharmaceutical Safety Division, Pharmaceutical Safety and Environmental Health Bureau of the Ministry of Health, Labor and Welfare. The PMDA also accepts consultations from marketing authorization holders (MAHs) on revisions of drug package inserts (PIs). The PMDA evaluates these post-marketing data and considers the need for safety measures. Based on these considerations, MAHs implement safety measures, such as PI revisions [1].

PI revisions are important safety measures and comprise the following steps: (1) information collection, (2) signal detection of adverse events (AEs), (3) signal validation, (4) signal evaluation, (5) consideration of safety measures based on risk classification, (6) expert discussion, and (7) implementation of safety measures. The first step is collecting ADR reports from MAHs and those reported by medical professionals, which are stored in the PMDA’s ADR database (information collection). The collected data are then subjected to signal detection, and the need for a signal evaluation is based on the drug’s safety profile, including PI descriptions, accumulated related reports, an index value of signals, the status of foreign regulatory authorities, and the results of signal detection and enhancement using medical information databases (signal validation). Then, secondary screening is conducted to assess whether the validated signal is a new risk (signal evaluation). The PMDA also consults with MAHs to determine if the risk is new and whether safety measures are necessary. If the PMDA finds insufficient evidence suggesting that the signal is a new risk or that the risk classification should change, the review process ends, and monitoring continues at the signal detection stage. In the signal evaluation stage, the PMDA determines the significance of the identified or potential risk. In cases where safety measures (such as a risk management plan revision, PI revision for risk mitigation, or provision of information to healthcare professionals) become necessary owing to the new risk classification, the PMDA will consider the need for expert discussion, draft safety measures, and an implementation strategy (including consideration of safety measures based on risk classification). The PMDA asks external experts to assess the appropriateness of safety decisions based on signal evaluation and risk classification (expert discussion). If the external expert panel determines that safety measures are necessary, they are implemented (implementation of safety measures) [2, 3].

In each step of the signal detection and validation, risky candidates are efficiently screened using disproportionality analyses [4, 5]. Disproportionality analyses, such as the proportional reporting ratio (PRR), the reporting odds ratio (ROR), and multi-item gamma Poisson shrinker, are used by regulatory authorities, in general [6–9]. Recently, methods using propensity scores or machine learning (ML)-based models have been proposed to improve signal detection performance over conventional disproportionality analysis based on a simple 2 × 2 contingency table [10–13]. By taking into account the quality of case reports and their content, these models have better predictive performance than the conventional disproportionality analysis as well as better interpretability of the detected signals. However, because signal detection methods do not directly indicate the need for safety measures, even with improved model performance, many steps, including communication with experts, confirmation, and communication with MAHs, are required before a decision is made to implement safety measures, which is time-consuming and costly. However, no model that directly predicts the need for safety measures has been reported. Hence, there is an urgent and unmet need for a tool that can quickly determine whether additional safety measures are needed. Such a tool might efficiently screen potential risk signals while supporting the safety measure decision-making process. Herein, we focused on adding a clinically significant adverse reaction (CSAR) section to the drug PIs as a revision in Japan and aimed to develop a ML-based prediction model for PI revisions at an early stage based on past information on ADRs.

## Materials and Methods

### Data Source and Study Population

In Japan, PIs are revised due to accumulated domestic, both domestic and overseas, overseas ADR cases, up-to-date company core data sheet, literature information, epidemiological information, request from academic societies, etc. Among them, literature information, epidemiological information and request from academic societies accounted for only 2.1% (13/618) of the reasons for adding CSARs to the PI during the analysis period. These impacts on the predictive model are expected to be minimal. Therefore, we focused on PI revisions between August 2011 and March 2020, which occurred due to the accumulation of domestic cases, the most common reason for adding CSARs to the PI in Japan, as the prediction target. The features used for the construction of prediction models were created based on the information recorded in the Japanese Adverse Drug Event Report (JADER) database.

The JADER database contains domestic ADR cases. The JADER contains four Table (1) DEMO (sex, age, weight, and other patient characteristics), (2) DRUG (drug characteristics, including name and other properties), (3) REAC (types of ADRs and their outcomes), and (4) HIST (medical history). The DRUG table includes information on suspected drugs, drug interactions, and concomitant medications. As the JADER contains up to 4 months old data, we used the data released in July 2020 for our model construction to track the addition of CSAR information to the PIs during March 2020. The JADER contains cases where the onset of ADRs were recorded prior to the initial administration of the suspected drug, cases where the same ADR was reported multiple times for the same patient, and records of ADRs for over-the-counter-drugs. These data were excluded from the study population because they were unsuitable for the purpose of predicting the addition of CSARs to PIs. Herein, each tabulation was performed only for the suspected drugs.

### Outcomes

**Figure 1.**
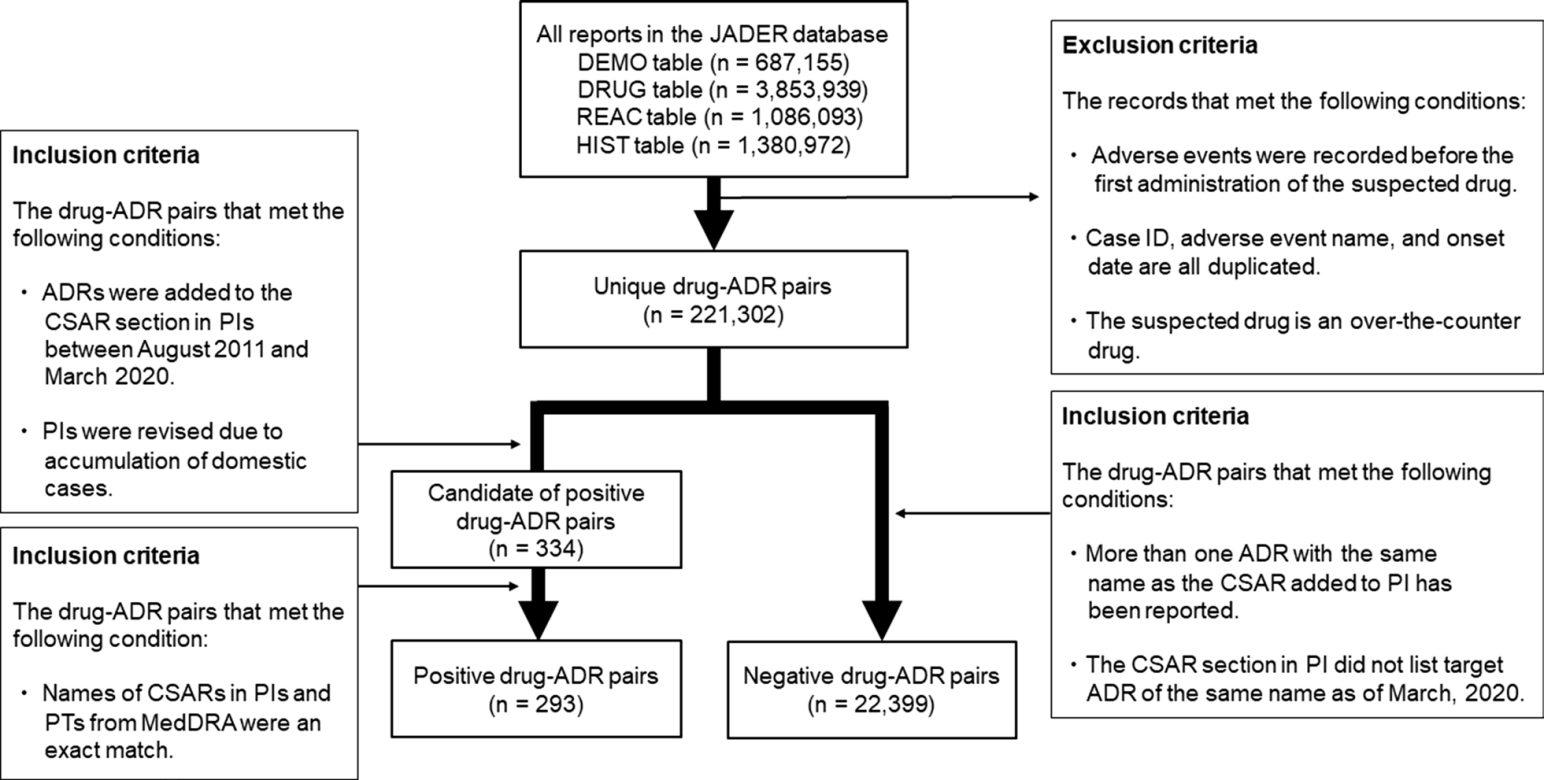
Data extraction scheme for positive and negative cases. The JADER database extracted 293 positive and 22,399 negative drug–ADR pairs. *JADER* Japanese Adverse Drug Event Report, *ADR* adverse drug reaction, *CSAR* clinically significant adverse reaction, *PI* package insert, *PT* preferred term, *MedDRA* medical dictionary for regulatory activities.

In this study, 75 different CSARs were used as predictors for which there was an exact match between the ADR name on the CSAR section in PI and preferred terms (PTs) from the Medical Dictionary for Regulatory Activities (MedDRA). PI revisions were not announced until weeks or months after the risk investigation started. Therefore, we used data from approximately two quarters before the PI revision, when CSAR information was added as positive cases to fill the gap. Negative cases included drug–ADR pairs that met the following criteria: (1) at least one of the 75 target CSARs was reported during the analysis period, and (2) the CSAR section in the PI did not list a target ADR with the same name as that listed on March 2020. The data-extraction scheme is illustrated in Fig. 1.

Each ADR was tabulated using PTs from the MedDRA version 23.0. For some ADRs, the PTs were grouped before each tabulation. PTs included in the target disease were calculated by referring to both the cumulative number of reported ADRs for each drug in the positive group and the Standardized MedDRA Queries. As symptoms may also be associated with other diseases, only the minimum necessary symptoms were included. For example, if there was a PI revision for “hyperkalemia,” cases of both hyperkalemia and increased blood potassium levels would be added to the hyperkalemia tally, and the characteristics would be tabulated. The grouping of each ADR is shown in Table 1.

**Table 1:** Grouping of adverse drug reactions based on the preferred terms.

Target disease	Preferred term
Interstitial lung disease	Interstitial lung disease, eosinophilic pneumonia, lung disorder, pneumonitis, organizing pneumonia, pulmonary fibrosis, idiopathic pulmonary fibrosis
Hepatic function abnormal	Hepatic function abnormal, liver disorder, drug-induced liver injury
Anaphylactic reaction	Anaphylactic shock, anaphylactic reaction, shock, blood pressure decreased, urticaria
Platelet count decreased	Thrombocytopenia, platelet count decreased
Rhabdomyolysis	Rhabdomyolysis
Erythema multiforme	Erythema multiforme
Toxic epidermal necrolysis	Toxic skin eruption, toxic epidermal necrolysis
Agranulocytosis	Agranulocytosis, granulocytopenia, and granulocyte count decreased
Hepatitis fulminant	Hepatitis fulminant
Ileus	Ileus, intestinal obstruction, ileus paralytic, mechanical ileus
Stevens–Johnson syndrome	Stevens–Johnson syndrome, oculomucocutaneous syndrome
Pancytopenia	Pancytopenia
Cardiac failure congestive	Cardiac failure congestive, cardiac failure
Acute generalized exanthematous pustulosis	Acute generalized exanthematous pustulosis
Acute kidney injury	Acute kidney injury
Pemphigoid	Pemphigoid
Hepatitis B reactivation	Hepatitis B, Hepatitis B DNA increased, Hepatitis B DNA assay positive, Hepatitis B virus test positive, Hepatitis B reactivation
Pancreatitis acute	Pancreatitis acute
Drug hypersensitivity	Drug reaction with eosinophilia and systemic symptoms, Drug hypersensitivity
Nephrotic syndrome	Nephrotic syndrome
Hypoglycemia	Blood glucose decreased, hypoglycemia, hypoglycemic seizure
Neuroleptic malignant syndrome	Neuroleptic malignant syndrome
Deep vein thrombosis	Deep vein thrombosis, pulmonary embolism
Hemophagocytic lymphohistiocytosis	Hemophagocytic lymphohistiocytosis
Shock symptom	Anaphylactoid shock, shock symptom
Hyponatremia	Blood sodium decreased, hyponatremia
Hypotension	Hypotension
Neutropenia	Neutrophil count decreased, neutropenia, autoimmune neutropenia, idiopathic neutropenia, febrile neutropenia
Tubulointerstitial nephritis	Tubulointerstitial nephritis
Ventricular tachycardia	Ventricular tachycardia, ventricular tachyarrhythmia
Depressed level of consciousness	Depressed level of consciousness, loss of consciousness, Altered state of consciousness, hallucination, Hallucination, visual
Cholangitis sclerosing	Cholangitis sclerosing
Tuberculosis	Tuberculosis
Intestinal perforation	Ileal perforation, large intestine perforation, intestinal perforation, rectal perforation
Colitis ischemic	Colitis ischemic
Guillain–Barre syndrome	Guillain–Barre syndrome
Ketoacidosis	Diabetic ketoacidosis, ketoacidosis
Metabolic acidosis	Metabolic acidosis
Hypomagnesemia	Hypomagnesemia
Ovarian hyperstimulation syndrome	Ovarian hyperstimulation syndrome
Stomatitis	Stomatitis
Laryngospasm	Laryngospasm
Enteritis	Enteritis
Acute respiratory distress syndrome	Acute respiratory distress syndrome
Acute disseminated encephalomyelitis	Acute disseminated encephalomyelitis
Cholecystitis acute	Cholecystitis acute
Inappropriate antidiuretic hormone secretion	Inappropriate antidiuretic hormone secretion
Disseminated intravascular coagulation	Disseminated intravascular coagulation
Sepsis	Bacterial sepsis, sepsis, septic shock
Neonatal cardiac failure	Cardiac failure high output, neonatal cardiac failure
Pneumothorax	Pneumothorax
Gastrointestinal perforation	Gastrointestinal perforation, upper gastrointestinal perforation, Lower gastrointestinal perforation
Hemolytic anemia	Autoimmune hemolytic anemia, hemolytic anemia
Meningitis aseptic	Meningitis aseptic
Hyperthyroidism	Hyperthyroidism
Hypothyroidism	Thyroiditis, hypothyroidism
Leukoencephalopathy	Posterior reversible encephalopathy syndrome, progressive multifocal leukoencephalopathy, leukoencephalopathy
Hepatic failure	Hepatic failure
Gastric antral vascular ectasia	Gastric antral vascular ectasia
Gastric ulcer	Gastroduodenal ulcer, Gastric ulcer, Duodenal ulcer, Gastric ulcer hemorrhage, Duodenal ulcer hemorrhage
Cerebral infarction	Thrombotic cerebral infarction, cerebellar infarction, cerebral infarction, hemorrhagic cerebral infarction, embolic cerebral infarction, ischemic cerebral infarction
Nephrogenic diabetes insipidus	Nephrogenic diabetes insipidus
Renal impairment	Renal impairment
Pyelonephritis	Pyelonephritis acute, pyelonephritis
Tumor lysis syndrome	Tumor lysis syndrome
Tendon rupture	Tendon rupture
Thrombotic microangiopathy	Thrombotic microangiopathy
Optic neuritis	Optic neuritis
Myasthenia gravis	Myasthenia gravis, myasthenia gravis crisis
Copper deficiency	Blood copper decreased, copper deficiency
Deafness	Mixed deafness, deafness neurosensory, deafness
Osteomalacia	Osteomalacia
Hyperkaliemia	Blood potassium increased, hyperkaliemia
Hyperglycemia	Hyperglycemia
Type 1 diabetes mellitus	Type 1 diabetes mellitus, fulminant type 1 diabetes mellitus

### Feature Data and Data Pre-processing

We created 34 features based on information that strengthens the signals written in CIOMS Working Group VIII and GVP Module VIII, information suggesting a causal relationship between the drug and the adverse event, and information that is focused on in pharmacovigilance activities at MAHs, including cumulative report counts that indicate the absolute amount of reporting, average number of missing values per case that indicate insufficient information about drugs or ADRs, number of re-administrations and recurrences suggesting the causality between drugs and ADRs, average and median number of days from drug administration to the onset of ADRs, and various disproportional reporting indicators. The disproportionality signal and the relative value compared to those of the other drug–ADR pairs were calculated as follows:

$${\text{ROR}}\; = \;\frac{{\left( {a/c} \right)}}{{\left( {b/d} \right)}} \; = \; \frac{ad}{{bc}}$$

$${\text{PRR}} = \frac{{{\text{a }}/{ }\left( {{\text{a }} + {\text{ c}}} \right)}}{{{\text{ b }}/{ }\left( {{\text{b }} + {\text{ d}}} \right)}} = \frac{{a\left( {b + d} \right)}}{{b\left( {a + c} \right)}}$$

$${\text{Pearson's}}\;{\text{Chi - squared}}\;{\text{test}}\;{\text{with}}\;{\text{Yates}}' \;{\text{continuity}}\;{\text{correction}}\; = \;\frac{{\left( {a + b + c + d} \right)(\left| {ad - bc} \right| - \left( {a + b + c + d} \right)/2)^{2} }}{{\left( {a + b} \right)\left( {c + d} \right)\left( {a + c} \right)\left( {b + d} \right)}}$$

$${\text{Index}}\;{\text{A}}\; = \;\frac{100 \times a}{{a + c}}$$

$${\text{Index}}\;{\text{B}}\; = \;\frac{100 \times a}{{a + b}}$$

here, *a*, *b*, *c*, and *d* are defined using the 2 × 2 table as follows:

*a*: the number of ADR cases that occurred after using the suspected drugs, b: the number of ADR cases that occurred after using all other drugs, c: the number of all other ADR cases that occurred after using the suspected drugs, and d: the number of all other ADR cases that occurred after using all other drugs. Table 2 lists the features generated based on the JADER data.

**Table 2:** All the features included in our dataset.

Feature name	Feature type
Number of patients	Int
Number of deaths	Int
Number of patients with drug re-administration	Int
Number of patients who discontinued the suspected drug	Int
The average number of days between administration of the suspect drug and the onset of the adverse drug reaction	Float
The median number of days between administration of the suspect drug and the onset of the adverse drug reaction	Float
Number of patients within 15 days from the administration of the suspected drug to the onset of the adverse drug reaction	Int
Number of patients within 30 days from the administration of the suspected drug to the onset of the adverse drug reaction	Int
Number of patients within 90 days from the administration of the suspected drug to the onset of the adverse drug reaction	Int
The average number of missing values per case	Float
The average number of reports to the regulatory authority per case	Float
Index A	Float
Index B	Float
Number of quarters that have elapsed since the first adverse event was reported	Int
Number of newly reported patients from a quarter ago	Int
Number of new patients reported since before 2 quarters	Int
Number of new patients reported since before 3 quarters	Int
Number of new patients reported since before 4 quarters	Int
Number of newly reported deaths from a quarter ago	Int
Number of newly reported patients with drug re-administration from a quarter ago	Int
Number of newly reported patients who discontinued the suspected drug a quarter ago	Int
Number of newly reported patients within 15 days from the administration of the suspected drug to the onset of the adverse drug reaction from a quarter ago	Int
Number of newly reported patients within 30 days from the administration of the suspected drug to the onset of the adverse drug reaction from a quarter ago	Int
Number of newly reported patients within 90 days from the administration of the suspected drug to the onset of the adverse drug reaction from a quarter ago	Int
The average number of patients reported per quarter	Float
PRR	Float
Log PRR	Float
The lower limit of the confidence interval for PRR	Float
The upper limit of the confidence interval for PRR	Float
YatesChisq	Float
ROR	Float
Log ROR	Float
The lower limit of the confidence interval for ROR	Float
The upper limit of the confidence interval for ROR	Float

*PRR* proportional reporting ratio, *ROR* reporting odds ratio, *YatesChisq* Pearson’s Chi-squared test with Yates’ continuity correction, *Index A* the ratio of the target adverse event number of specific drug to all adverse event numbers that were reported for the same drug, *Index B* ratio of adverse events covered by a particular drug to those covered by all drugs

Before data aggregation, we excluded cases (1) included in JADER for which the ADR onset date preceded the first dose of the suspected drug; (2) with duplicate records with matching case IDs, ADR names, and onset dates; and (3) where the suspected drug was an over-the-counter drug (Fig. 1).

There were missing data for the date of drug administration and ADR occurrence, which resulted in 4,920 (21.7%) missing values in the “mean number of days from administration to occurrence” and “median number of days from administration to occurrence.” The “median number of days from administration to the onset of the same ADR for other prescription drugs” was used as a substitute in some models to address this issue. There were no missing data points for the other features. Each feature was transformed using standardization or quantile transformation, depending on the model used.

### Model Development and Performance Evaluation

To predict the addition of CSAR information to PIs, we developed classification models using eXtreme gradient boosting (XGBoost) [14], light gradient boosting machine (LightGBM) [15], and support vector machine (SVM) with a radial basis function kernel (RBF–SVM) [16–21].

As some features may not contribute to the prediction, we created a model trained with all the features and another trained with selected features using exhaustive feature selection (EFS). This EFS algorithm is a wrapper approach for brute-force evaluation of feature subsets; the best subset is selected by optimizing a given performance metric given an arbitrary regressor or classifier [22]. The dataset was divided 7:3 into training and test datasets. Hyperparameter optimization was performed on the model with the highest average training score using Optuna optimization with the hyperband method, and various hyperparameter combinations were tested [23]. The Matthews correlation coefficient (MCC) was used as the model evaluation metric to measure the accuracy of a binary classification model, which is considered a balanced measure that can be used when class sizes vary. The MCC ranged from − 1 to + 1, with + 1 representing a perfect prediction and 0 representing an average random prediction. As adding CSAR information to the PI is rare and the dataset is unbalanced, we evaluated the model’s performance using MCC.

$${\text{MCC }} = \frac{{\left( {{\text{TP}} \times {\text{TN}}} \right) - \left( {{\text{FP }} \times {\text{ FN}}} \right)}}{{\sqrt {\left( {{\text{TP }} + {\text{FP}}} \right) \times \left( {{\text{TP}} + {\text{FN}}} \right){ } \times \left( {{\text{TN}} + {\text{FP}}} \right){ } \times \left( {{\text{TN}} + {\text{FN}}} \right)} }}$$

TP, true positive; TN, true negative; FP, false positive; FN, false negative.

All modeling and calculations were performed using Python version 3.9.3.

## Results

### Baseline Characteristics

Of all the PI revisions during the analysis period, 54% (334 cases) were due to accumulated domestic cases. Among the 334 cases, 293 drug-ADR pairs were identified in which the ADR name in the CSARs section of the PI matched that in the PTs in MedDRA.

As shown in Fig. 1, 221,302 unique drug-ADR pairs were identified as the pairs that met the definition of positive and negative cases. Finally, 293 positive and 22,399 negative cases were used for model development and evaluation. The positive group had fewer missing values per case and more regulatory authority reports per case than the negative group. Few patients were re-administered the suspected drug, and the variability of each feature was high [Electronic Supplementary Material (ESM) Table S1].

### Construction of the ML-Based Prediction Models and Comparison of Six Models

**Figure 2:**
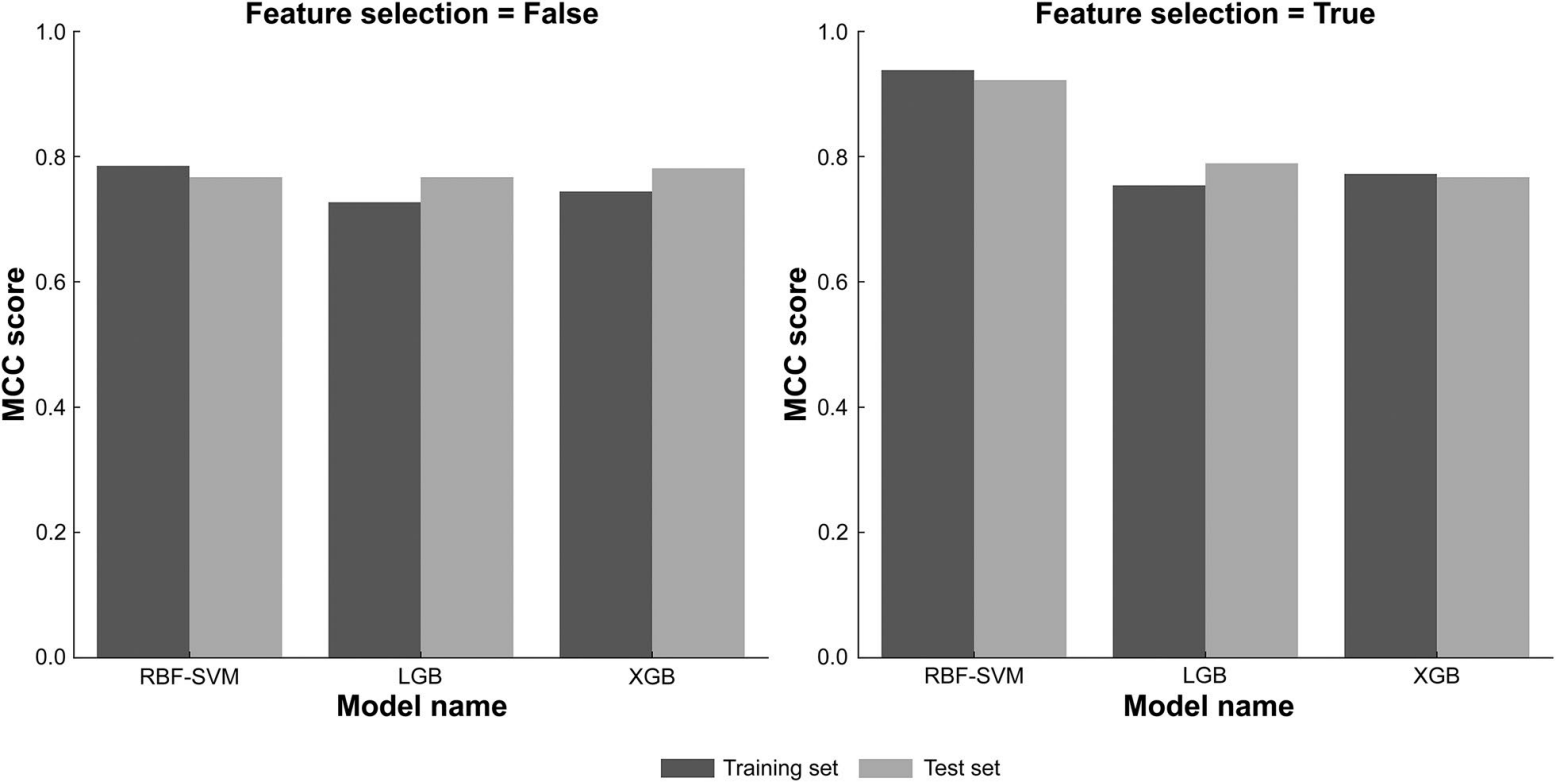
The MCC scores for each machine-learning model, with and without feature selection. *MCC* Matthews correlation coefficient; *RBF–SVM* support vector machine with radial basis function kernel; *LGB* light gradient-boosting machine; *XGB* eXtreme gradient boosting.

While not directly predicting the addition of CSAR information to PIs, a study that investigated improvements to statistical signal detection methods reported that the number of reports containing sufficient information about drugs and ADRs, the number of reports from the past 3 years, and disproportional reporting contributed to the improvement of signal detection methods [13]. Another study that investigated the signal characteristics associated with PI updates reported that drug age, mechanistic plausibility, seriousness of the event, and the confirmation of the signals in multiple types of data sources might predict ADRs requiring PI updates [24]. Therefore, we created features similar to those examined in previous studies that can be created with the information contained in JADER, such as the average number of missing data per case as sufficient information about drugs and ADRs, and the number of deaths reported for that drug-ADR pair as the seriousness of the event. To determine how far in advance of the revision the number of reported patients contributes to the prediction, the number of recent reports, which were determined to be of high importance in previous studies, was incorporated into the features at finer time intervals. For disproportional reporting, several indicators were added as features to determine which signal indicators contributed to the prediction. As we aimed to build a highly versatile forecasting model, we excluded information unique to the ADR or product (such as names of the ADRs or MAH information) from the features. Using these features, we constructed 6 prediction models using different ML algorithms. The prediction performance of each model is shown in Fig. 2 and ESM Table S2. The RBF–SVM model had the fewest false positives and negatives and the highest MCC. The RBF–SVM model with feature selection had the best prediction performance, with an MCC of 0.938 for cross-validation and 0.922 for the test data. Bayesian optimization of the RBF–SVM model with the best prediction performance was performed in the search range of C 0.01–3000 and gamma 0.001–1000 for 5000 iterations, and the MCC was 0.941 (*C* = 2388, gamma = 4.718) (Table 3).

**Table 3:** Prediction performance of the RBF–SVM model.

		Training data				Test data			
Model	Feature type	MCC	Precision	Recall	AUC	MCC	Precision	Recall	AUC
RBF–SVM	All	0.785 (0.044)	0.899 (0.066)	0.693 (0.068)	0.966 (0.024)	0.767	1.0	0.591	0.983
RBF–SVM	EFS	0.938 (0.032)	0.989 (0.019)	0.892 (0.047)	0.989 (0.012)	0.922	0.987	0.864	0.998
RBF–SVM (Optimized)	EFS	–	–	–	–	0.941	0.988	0.898	0.999

*RBF–SVM* support vector machine with the radial basis function kernel, *EFS* exhaustive feature selection, *MCC* Matthews correlation coefficient, *AUC* area under the curve

**Figure 3:**
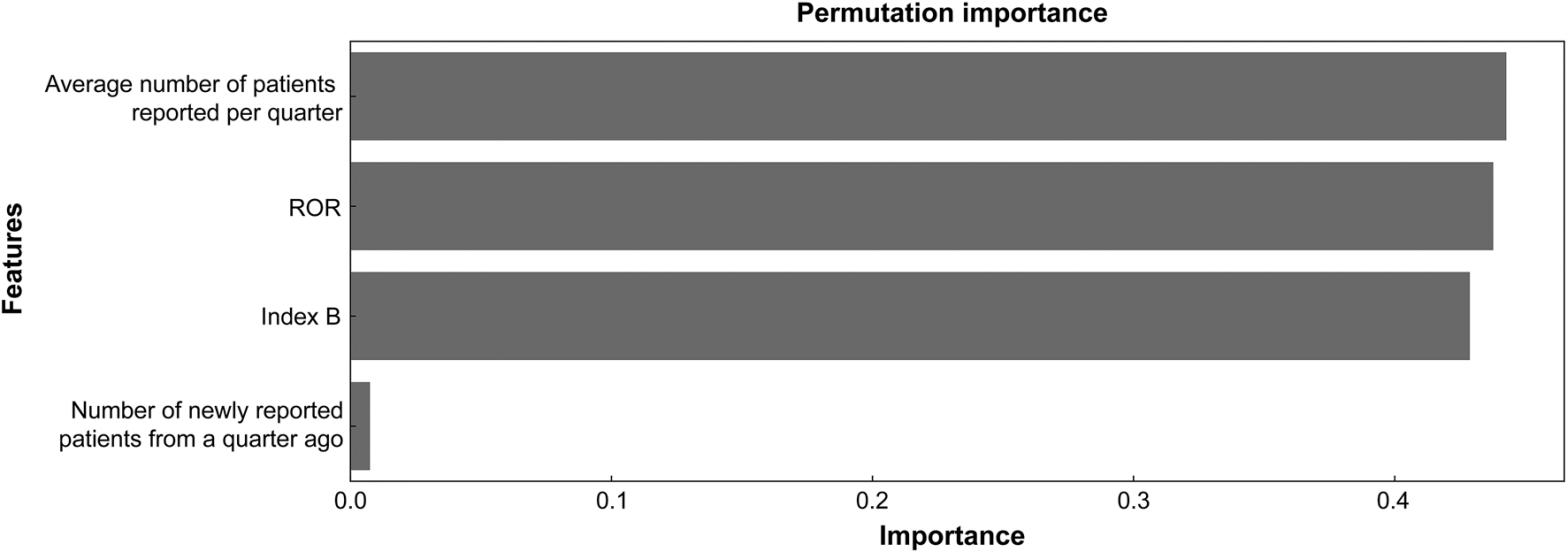
Permutation importance in the optimized RBF–SVM model. *ROR* reporting odds ratio; *Index B* ratio of adverse events covered by a particular drug to those covered by all drugs.

The permutation importance results from the optimized RBF–SVM model are shown in Fig. 3 [25]. A feature was considered significant if swapping its values increased the model’s error, indicating that the predictions depended on this feature. The feature with the highest importance in the model was the “average number of patients reported per quarter.”

### The Relationship Between Conventional Disproportionality Analysis and the Revision of PIs

To compare the prediction performance of our ML-based model, we analyzed the relationship between PRR as representative of a conventional disproportionality analysis and the revision of PIs. We used the PRR thresholds commonly applied in signal detection, namely a cumulative number of reports ≥ 3, a Chi-square value ≥ 4, and a PRR ≥ 2. Table 4 shows that PRR identified a high number of false positives, and only 4% (35/859) of the detected signals led to revisions of the PI, and it missed 53 cases (60.2%) that required revisions to the PIs.

**Table 4:** Relationship between the revision of PIs and PRR.

		Revision of PIs	
		Yes	No
Detection of signal	Yes	35	824
	No	53	5896

*PRR* proportional reporting ratio, *PI* package insert

## Discussion

To the best of our knowledge, this is the first study to predict the addition of CSAR information to PIs in Japan. The RBF–SVM model exhibited the best prediction performance, with an MCC of 0.941. The four features used in this model were “index B,” “the number of newly reported patients from a quarter ago,” “the average number of patients reported per quarter,” and “ROR.” The result of EFS confirmed that the selected features were not specific to RBF–SVM. “Index B,” “the number of newly reported patients from a quarter ago,” and “the average number of patients reported per quarter” were the most commonly reported features, even though different ML algorithms were used (ESM Table S3). Therefore, the significance of factors that predict the addition of CSAR information to PIs is consistent and independent of ML algorithms. This study is a binary classification that predicts whether CSARs are added to the PI or not. And with very high predictive performance, we were able to correctly predict positive and negative cases. Thus, we believe that the significant features identified in this study are crucial for predicting the absence of a change in CSAR section in PI, particularly when the prediction probability falls below a certain threshold. However, the negative cases selected this time meet certain criteria; therefore, a further study is needed to predict all instances of the absence of a change in the CSAR section in PI.

Rechallenge information and time to the onset of ADR, which indicate a causal relationship between a drug and an ADR and are expected to have a significant impact on the PI revisions [26], were uncritical predictive factors. Moreover, the “number of patients,” often used by MAHs as a criterion when considering the need for safety measures, was also insignificant, highlighting the need to pay attention to other features. A previous study aimed at improving the signal detection method, re-challenge, time to onset of ADR, and number of patients, which were of low importance, while recent reporting and disproportional reporting were of high importance [13]. Considering that signal detection is the first step in determining whether or not safety measures are necessary and that some of the detected signals are finally used to determine whether safety measures are necessary, it is reasonable to assume that similar features are important, although they are used for different purposes. Among the potential features that were not used in our study, there might be some that could improve the model's performance. However, we utilized the spontaneous reporting database, a crucial source of information for safety monitoring activities, and generated as many meaningful features as possible from it. As a result, we were able to construct a model that demonstrated extremely high predictive performance solely based on the information obtained from JADER. The fact that we were able to construct a high-performing predictive model using only a single database will benefit both the PMDA and MAHs. This is because the model is easy to use and will streamline their safety monitoring activities.

Contrary to our initial expectations, the RBF–SVM model performed the best. The RBF–SVM uses a kernel trick to map features onto a higher-dimensional space for linear analysis [21]. The linear analysis uses all the features in the model to make predictions, including the irrelevant features, and reduces the performance. The features of our model were cleanly and linearly separated, which might explain why combining the RBF–SVM and EFS met our objectives.

Although we calculated the prediction performance with a 0.5 threshold, the prediction probability for nearly all true-negative samples was in the range of 0–0.1. When the threshold was set to 0.1, the prediction performance improved with MCC 0.971, precision 0.988, recall 0.955, and AUC 0.999. Creating predictive models for imbalanced data often involves weighting, moving the threshold, and resampling [27]. As this study obtained a relatively high prediction performance by moving the threshold, future studies should determine the optimal threshold.

The ML-based prediction model can support efficient decision-making by shortening the time to consider whether or not to revise the PI. While signal detection screening increases workflow efficiency, it only screens for potential risk candidates among multiple drug–ADR pairs and has no role beyond prioritizing the signals [5, 7, 28]. Among the 1,888 safety signals detected in 2020, only 39 (2.1%) were validated by the European Medicines Agency’s Pharmacovigilance Risk Assessment Committee [29]. Safety signals detected using the signal detection method rarely changed the safety measures. To ascertain whether the data used in this study show a similar trend, we conducted a comparative analysis with conventional disproportionality analysis to determine its ability to detect signals that could potentially lead to the revision of PI (Table 4). The data used in this study also showed that many of the signals detected by the conventional disproportionality analysis did not influence the revision of the PIs. Although the conventional disproportionality analysis has played a role in capturing a wide range of risk candidates, it still has the problem of having to investigate a large number of false positives. The process leading to the decision to revise the PI involves several steps, including signal evaluation, validation, and expert review. Streamlining this process can help focus on critical signals that require evaluation. Our model directly predicted revisions to the PI and demonstrated good predictive performance with few false positives (1 case) and false negatives (4 cases). The target audience for this model comprises the PMDA and MAHs. We advocate for the utilization of conventional signal detection to widely identify risk candidates, and employ this model to pinpoint drug-ADR pairs that necessitate inclusion in the PI as CSARs during their routine safety measures evaluation and before the expert discussion. By incorporating this method into the standard workflow, they can efficiently determine whether safety measures are necessary while saving time and human resources. Additionally, to the best of our knowledge, this is the first study to develop an ML-based model for identifying the factors that affect the need for safety measures. PMDA and MAHs should consider these factors when determining appropriate safety measures.

This study has three limitations. The first is the data used. The model covered only the domestic (not overseas) CSARs that occurred during the study period. While most spontaneous reports in Japan are from healthcare professionals, reports from overseas include patient-reported reactions. Additionally, we do not know how healthcare system differences affect the predictions. Given the large number of reports from healthcare professionals, we limited this study to Japanese data to create a high-quality prediction model. However, more than half of PI revisions to add new CSARs in Japan were due to the accumulation of domestic cases. Therefore, this predictive model, which can accurately predict more than half of these cases, can significantly increase the efficiency of the signal management workflow. The second is the grouping of the CSARs. Our study’s grouping method can be used when the MedDRA-PT and PI names are exactly matched, but not when the PI name does not match the PT. In such cases, it is necessary to consult with medical experts or consider the accumulated ADRs of similar drugs before grouping. Regarding this limitation, as noted in the inclusion criteria in Fig. 1, although there were 41 cases affected by this limitation, approximately 88% (293 cases) of the PI additions of CSARs due to the accumulation of domestic cases fall within the scope of this predictive model. As it shows a high predictive performance in most cases, we believe that it can contribute to the efficiency of the signal management workflow. The third limitation is the predictable timing. The model was developed based on the hypothesis that predictions can be made based on information available approximately 6 months before the addition of CSAR information to PIs. However, if there is a high incidence of fatal side effects immediately after launch, CSAR information may be added to the PIs without waiting for 6 months, and our model would not detect these CSARs. The fourth is.

## Conclusion

The addition of CSAR information to PIs can be predicted directly based on past ADR information. Using JADER and ML, our model provides an efficient method to help decide the need for the implementation of safety measures.

### Supplementary Information

Below is the link to the electronic supplementary material.

Supplementary file1 (PDF 149 kb)

## Data Availability

Publicly available datasets were analyzed herein. These data can be found at https://www.pmda.go.jp/safety/info-services/drugs/adr-info/suspected-adr/0005.html.
