# Supplementary material for: Predicting the Addition of Information Regarding Clinically Significant Adverse Drug Reactions to Japanese Drug Package Inserts Using a Machine-Learning Model
Source: Ther Innov Regul Sci. 2023 Dec 22;58(2):357–67. doi: 10.1007/s43441-023-00603-4 (PMC10850196; doi:10.1007/s43441-023-00603-4)
Supplement: Supplementary file 1 — Supplementary file1 (PDF 149 kb) [file 43441_2023_603_MOESM1_ESM.pdf]

Table S1. Characteristics between the positive and negative groups

	Positive cases mean (std)	Negative cases mean (std)
Number of patients, n	12.4 (19.6)	7.5 (44.4)
Number of deaths, n	0.3 (1.3)	0.2 (1.8)
Number of patients with drug re-administration, n	0.0 (0.1)	0.0 (0.3)
Number of patients who discontinued the suspected drug, n	6.6 (13.4)	2.6 (22.3)
The average number of days between administration of the suspect drug and the onset of the adverse drug reaction, days	117.5 (254.0)	123.1 (331.0)
The median number of days between administration of the suspect drug and the onset of the adverse drug reaction, days	87.9 (267.8)	105.3 (325.0)
Number of patients within 15 days from the administration of the suspected drug to the onset of the adverse drug reaction, n	3.3 (10.7)	1.4 (23.6)
Number of patients within 30 days from the administration of the suspected drug to the onset of the adverse drug reaction, n	4.1 (11.2)	1.8 (26.0)
Number of patients within 90 days from the administration of the suspected drug to the onset of the adverse drug reaction, n	5.5 (12.0)	2.5 (29.7)
The average number of missing values per case, n	3.9 (1.5)	5.5 (3.0)

The average number of reports to the regulatory authority per case, n	3.1 (1.5)	2.4 (1.2)
Index A	3.9 (7.3)	2.5 (6.8)
Index B	1.6 (5.9)	0.2 (0.9)
Number of quarters that have elapsed since the first adverse event was reported, quarters	17.8 (13.5)	36.7 (20.4)
Number of newly reported patients from a quarter ago, n	1.2 (1.9)	0.0 (0.0)
Number of new patients reported since before 2 quarters, n	2.1 (2.9)	0.2 (1.5)
Number of new patients reported since before 3 quarters, n	3.0 (4.0)	0.3 (2.2)
Number of new patients reported since before 4 quarters, n	3.7 (4.9)	0.4 (2.8)
Number of newly reported deaths from a quarter ago, n	0.1 (1.0)	0.0 (0.0)
Number of newly reported patients with drug re-administration from a quarter ago, n	0.0 (0.2)	0.0 (0.0)
Number of newly reported patients who discontinued the suspected drug from a quarter ago, n	0.6 (4.3)	0.0 (5.0)
Number of newly reported patients within 15 days from the administration of the suspected drug to the	−0.5 (8.2)	0.0 (0.2)

onset of the adverse drug reaction from a quarter ago, n		
Number of newly reported patients within 30 days from the administration of the suspected drug to the onset of the adverse drug reaction from a quarter ago, n	−0.5 (8.2)	0.0 (0.2)
Number of newly reported patients within 90 days from the administration of the suspected drug to the onset of the adverse drug reaction from a quarter ago, n	−0.6 (8.7)	0.0 (0.3)
The average number of patients reported per quarter, n	11.8 (16.5)	14.4 (24.4)
PRR	49.0 (423.7)	6.3 (63.9)
LogPRR	0.8 (1.8)	0.2 (1.5)
The lower limit of the confidence interval for PRR	21.0 (178.1)	2.1 (40.7)
The upper limit of the confidence interval for PRR	124.5 (1036.1)	26.7 (197.8)
YatesChisq	248.8 (1645.3)	65.8 (2518.4)
ROR	57.9 (530.8)	8.1 (109.8)
LogROR	0.9 (1.8)	0.3 (1.6)
The lower limit of the confidence interval for ROR	22.2 (191.6)	2.2 (60.2)
The upper limit of the confidence interval for ROR	163.7 (1494.8)	51.7 (706.4)

PRR, proportional reporting ratio; ROR, reporting odds ratio; YatesChisq, Pearson's chi-squared test with Yates' continuity correction; Index A, the ratio of the target adverse event number of specific drug to all adverse event numbers that were reported for the same drug; Index B, ratio of adverse events covered by a particular drug to those covered by all drugs

Table S2. Prediction performance of all models

	Model	Feature Type	Validation MCC	Validation Precision	Validation Recall	Validation AUC	Test data MCC	Test data Precision	Test data Recall	Test data AUC
1	SVM - RBF Kernel	All	0.785 (0.044)	0.899 (0.066)	0.693 (0.068)	0.966 (0.024)	0.767	1.000	0.591	0.983
2	Extreme Gradient Boosting	All	0.744 (0.093)	0.981 (0.033)	0.575 (0.128)	0.787 (0.064)	0.781	1.000	0.614	0.807
3	Light Gradient Boosting	All	0.727 (0.099)	0.960 (0.042)	0.560 (0.125)	0.780 (0.063)	0.767	0.981	0.602	0.801
4	SVM - RBF Kernel	EFS	0.938 (0.032)	0.989 (0.019)	0.892 (0.047)	0.989 (0.012)	0.922	0.987	0.864	0.998
5	Extreme Gradient Boosting	EFS	0.772 (0.078)	0.973 (0.035)	0.619 (0.10	0.809 (0.05	0.767	0.981	0.602	0.801

					1)	1)				
6	Light Gradient Boosting	EFS	0.754 (0.078)	0.954 (0.042)	0.604 (0.113)	0.802 (0.056)	0.789	1.000	0.625	0.812

RBF–SVM, support vector machine with the radial basis function kernel; EFS, exhaustive feature selection; MCC, Matthews correlation coefficient; AUC, area under the curve

Table S3. Selected features in each model

Model	Selected features
RBF-SVM	・Index B
	・Number of newly reported patients from a quarter ago
	・Average number of patients reported per quarter
	・ROR
Light Gradient Boosting	・Index B
	・Number of quarters that have elapsed since the first adverse event was reported
	・Number of newly reported patients from a quarter ago
	・Average number of patients reported per quarter
	・ Upper limit of the confidence interval of ROR
Extreme Gradient Boosting	・Number of patients
	・Index B
	・Number of newly reported patients from a quarter ago
	・Average number of patients reported per quarter
	・Lower limit of the confidence interval of PRR
	・Upper limit of the confidence interval of PRR

PRR, proportional reporting ratio; ROR, reporting odds ratio; Index A, the ratio of the target adverse event number of specific drug to all adverse events numbers that reported for the same drug; Index B, ratio of adverse events covered by a particular drug to those covered by all drugs
